# The RBP1–CKAP4 axis activates oncogenic autophagy and promotes cancer progression in oral squamous cell carcinoma

**DOI:** 10.1038/s41419-020-2693-8

**Published:** 2020-06-25

**Authors:** Ling Gao, Qibo Wang, Wenhao Ren, Jingjing Zheng, Shaoming Li, Zhichao Dou, Xinjuan Kong, Xiao Liang, Keqian Zhi

**Affiliations:** 1 Department of Oral and Maxillofacial Surgery, the Affiliated Hospital of Qingdao University, Qingdao, Shandong China; 2 Key Laboratory of Oral Clinical Medicine, the Affiliated Hospital of Qingdao University, Qingdao, Shandong China; 3 Department of Basic & Translational Sciences, University of Pennsylvania, School of Dental Medicine, Philadelphia, PA 19104 USA; 4 Department of Stomatology, the Affiliated Hospital of Qingdao University, Qingdao, Shandong China; 5 Department of Gastroenterology, the Affiliated Hospital of Qingdao University, Qingdao, Shandong China; 6 Department of Neurology, Haukeland University Hospital, 5021 Bergen, Norway

**Keywords:** Protein-protein interaction networks, Oral cancer

## Abstract

Retinol-binding protein 1 (RBP1) is involved in several physiological functions, including the regulation of the metabolism and retinol transport. Studies have shown that it plays an important role in the pathogenesis of several types of cancer. However, the role of RBP1 and its correlation with autophagy in oral squamous cell carcinoma (OSCC) pathogenesis remain unknown. In this study, RBP1 was identified as the most significantly upregulated DEPs with a >2-fold change in OSCC samples when compared to normal tissues through iTRAQ-based proteomics analysis coupled with 2D LC–MS/MS. RBP1 overexpression was significantly associated with malignant phenotypes (differentiation, TNM stage, and lymphatic metastasis) of OSCC. In vitro experiments demonstrated that RBP1 was significantly increased in OSCC tissues and cell lines compared with control group. RBP1 overexpression promoted cell growth, migration, and invasion of OSCC cells. Silencing of RBP1 suppressed tumor formation in xenografted mice. We further demonstrated that the RBP1–CKAP4 axis was a critical regulator of the autophagic machinery in OSCC, inactivation of autophagy rescued the RBP1–CKAP4-mediated malignant biological behaviors of OSCC cells. Overall, a mechanistic link was provided by RBP1–CKAP4 between primary oncogenic features and the induction of autophagy, which may provide a potential therapeutic target that warrants further investigation for treatment of OSCC.

## Introduction

Oral squamous cell carcinoma (OSCC) is one of the most common malignant tumors and accounts for ~90% of oral cancers^1,2^. Mortality and recurrence rate of OSCC has not decreased significantly during the past 30 years despite advances in therapeutic strategies and 5‐year survival rate remains <50% (ref. ^3,4^). More than 60% of OSCC patients are diagnosed at advanced stages (III and IV). The survival rate and morbidity due to OSCC would be dramatically improved if it could be detected and intervened at an early stage. Although multiple studies have been carried out to analyze the pathogenesis of OSCC, its biology and pathology are still not clearly understood.

Retinoids suppress carcinogenesis and regulate the growth, differentiation, and apoptosis of normal cells during embryonic development, as well as of premalignant and malignant cells^5^. Retinoic acid is reported to be present in the circulation, but most tissues rely on the uptake and cytosolic metabolism of retinoic acid to activate retinoic acid receptors (RARs) and retinoid X receptors^6,7^. Retinol-binding protein 1 (RBP1) is a cytosolic carrier that is responsible for regulating retinol homeostasis in various human and rodent tissues^8,9^. It possesses high‐affinity binding for retinoic acid and possibly functions as a chaperone‐like protein to regulate the prenuclear phase of retinoic acid signaling^10–12^. Studies have reported that RBP1 is abnormally expressed in several human cancers, including OSCC (refs. ^13–16^). However, the role and molecular mechanisms of RBP1 in the pathogenesis of OSCC remain largely unclear.

Cytoskeleton-associated protein 4 (CKAP4, also known as P63, CLIMP-63, and ERGIC-63) is a type II transmembrane protein that is reversibly palmitoylated^17,18^. It is originally localized to the ER and binds to microtubules. CKAP4 is subsequently discovered in the cell surface membrane of several cell types, including bladder epithelial cells, vascular smooth muscle cells, and type II pneumocytes. It functions as a receptor for several ligands, including anti-proliferating factor, surfactant protein A, and tissue plasminogen activator^19–21^. CKAP4 is also recognized as a novel Dickkopf1 (DKK1)-interacting protein, which was originally identified as an embryonic head inducer in *Xenopus* embryos and a downstream target gene of the β-catenin pathway^22^. Previous study has demonstrated that CKAP4-mediated DKK1 signaling regulated cancer cell growth via PI3K/AKT pathway^23^.

Autophagy serves as a dynamic degradation and recycling system, and it provides biological materials and energy in response to stress. Autophagy has a complex role in the pathogenesis of cancer and its function can be dependent on biological factors, such as the tumor type, driving the oncogenes and tumor suppressor genes to either inhibit or stimulate tumorigenesis, indicating that autophagy has opposing, context-dependent roles in cancer^24^. Thus, it is required to explore the potential roles of RBP1 and autophagy in the progression of OSCC, RBP1-related targeting autophagy might become a novel approach in cancer therapy. Recently, an innovative technology, isobaric tags for relative and absolute quantitation (iTRAQ) in conjunction with two-dimensional liquid chromatography and tandem mass spectrometry (2D LC–MS/MS) analysis has been used to identify candidate biomarkers in several cancers, including colon cancer^25^, breast cancer^26^, and cholangiocarcinoma^27^. Our group has previously demonstrated the use of this technique for detection of proteins with high molecular weight proteins that are seriously acidic or basic or proteins, which reside in the cell membrane^28^.

In this study, we performed iTRAQ and then screened 893 upregulated and 358 downregulated DEPs enriched from OSCC samples compared to paired normal tissues. We identified the upregulation of RBP1 in OSCC tissues. RBP1 overexpression promoted cell growth, migration, and invasion of SCC15 cells in vitro. Silencing of RBP1 in SCC15 cells suppressed tumor formation in vivo. More importantly, we further identified that RBP1–CKAP4 axis was a critical regulator of autophagic machinery in OSCC. Collectively, results from our study suggested that RBP1 could be a potential biomarker for OSCC patients and that RBP1-induced autophagy via CKAP4 axis might be a potential target for the treatment of OSCC.

## Results

### RBP1 was increased in OSCC tissues with positive correlation with malignant degree of OSCC patients

**Fig. 1:**
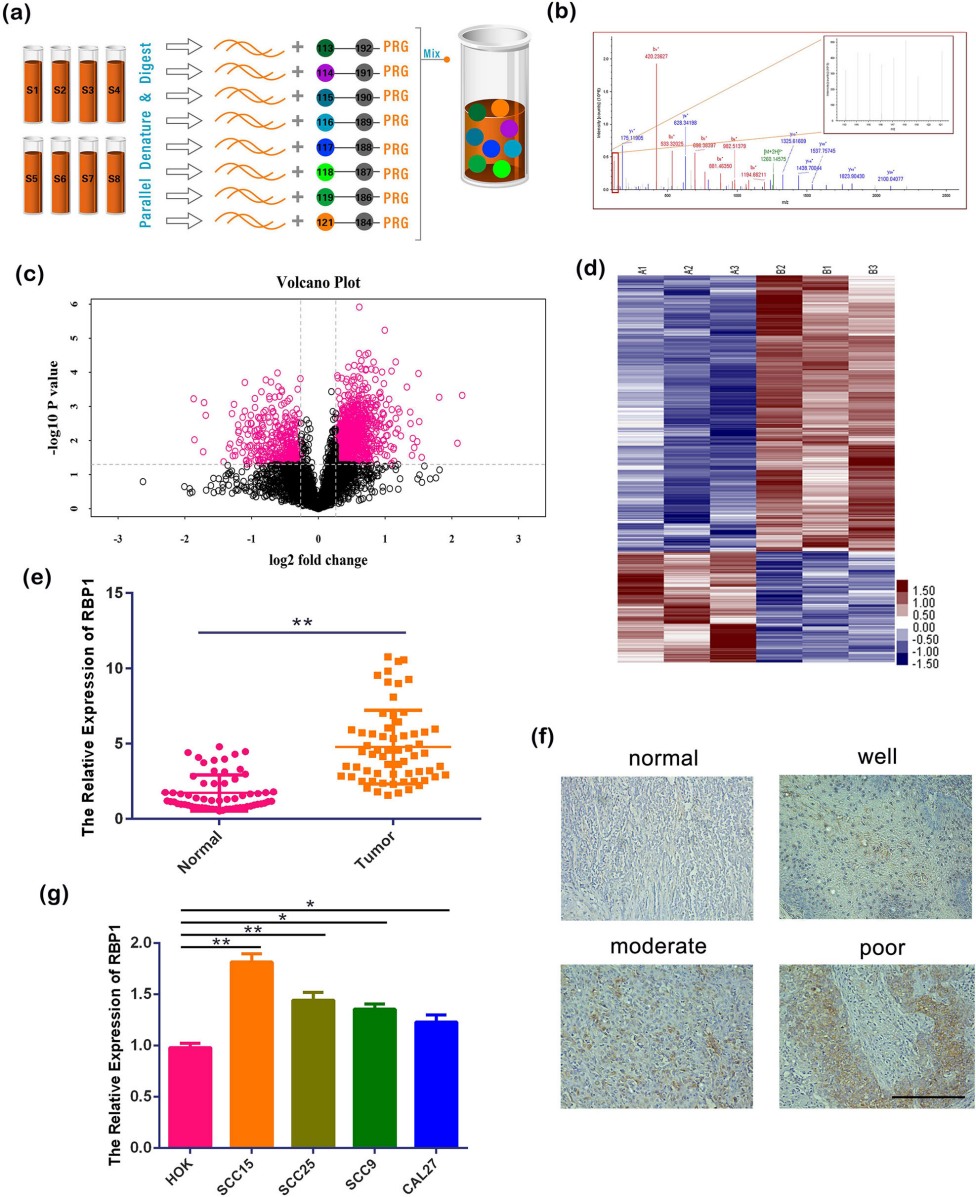
The expression of RBP1 in patients with OSCC and cell lines. **a**, **b** iTRAQ technical flow chart and mass spectrometry map of a DEP. **c**, **d** Volcanic maps and cluster analysis of DEPs. **e** The expression of RBP1 in 63 pairs of OSCC tissues and normal tissues were analyzed by qRT-PCR. **f** IHC was used to analyze the expression of RBP1 in 63 pairs of OSCC tissues with different degrees of differentiation and normal tissues. Scale bar: 100 μm. **g** The expression of RBP1 in OSCC cell lines and HOK cell were analyzed by qRT-PCR. All data were shown as mean ± SD; *P < 0.05, **P < 0.01.

We performed iTRAQ combined with 2D LC–MS/MS with three paired OSCC and normal tissues to identify the potential tumor biomarkers in OSCC. Using the ProteinPilot software and Volcano Plot analysis, 893 upregulated and 358 downregulated DEPs were screened (Fig. 1a–d). RBP1 was identified as one of the most significant upregulated DEPs with fold change >2 and *P* < 0.01 in OSCC samples compared to normal tissues. To further validate the results of iTRAQ, a total of 63 OSCC patients were enrolled with inclusion criteria of primary disease, no systemic disease, no history of smoking or drinking, and no received treatments. We then validated the result that RBP1 was increased in OSCC compared with normal tissues using quantitative real-time PCR (qRT-PCR) analysis (Fig. 1e). In addition, we performed the immunohistochemistry (IHC) staining and found that the RBP1 expression was consistent with that of qRT-PCR (Fig. 1f). A significant positive relationship between RBP1 expression level and poor differentiation, advance stage and lymphatic metastasis was seen when statistical analysis was done (Supplementary Table 1).

To confirm the RBP1 upregulation in cellular level, we performed qRT-PCR to analyze the gene expression level of RBP1 in four different OSCC cell lines, including SCC15, SCC25, SCC9, and CAL27. Human oral keratinocyte (HOK) was used as a normal control. Consistent with the findings from patient tissues, the above results demonstrated that there was a significant increase of RBP1 expression in four OSCC cell lines compared with HOK, especially in SCC15 cells. (Fig. 1g, *P* < 0.05). These results suggested that RBP1 can be considered for assembly into candidate biomarker in OSCC and investigated in the further studies.

### RBP1 overexpression promoted cell growth, migration, and invasion

**Fig. 2:**
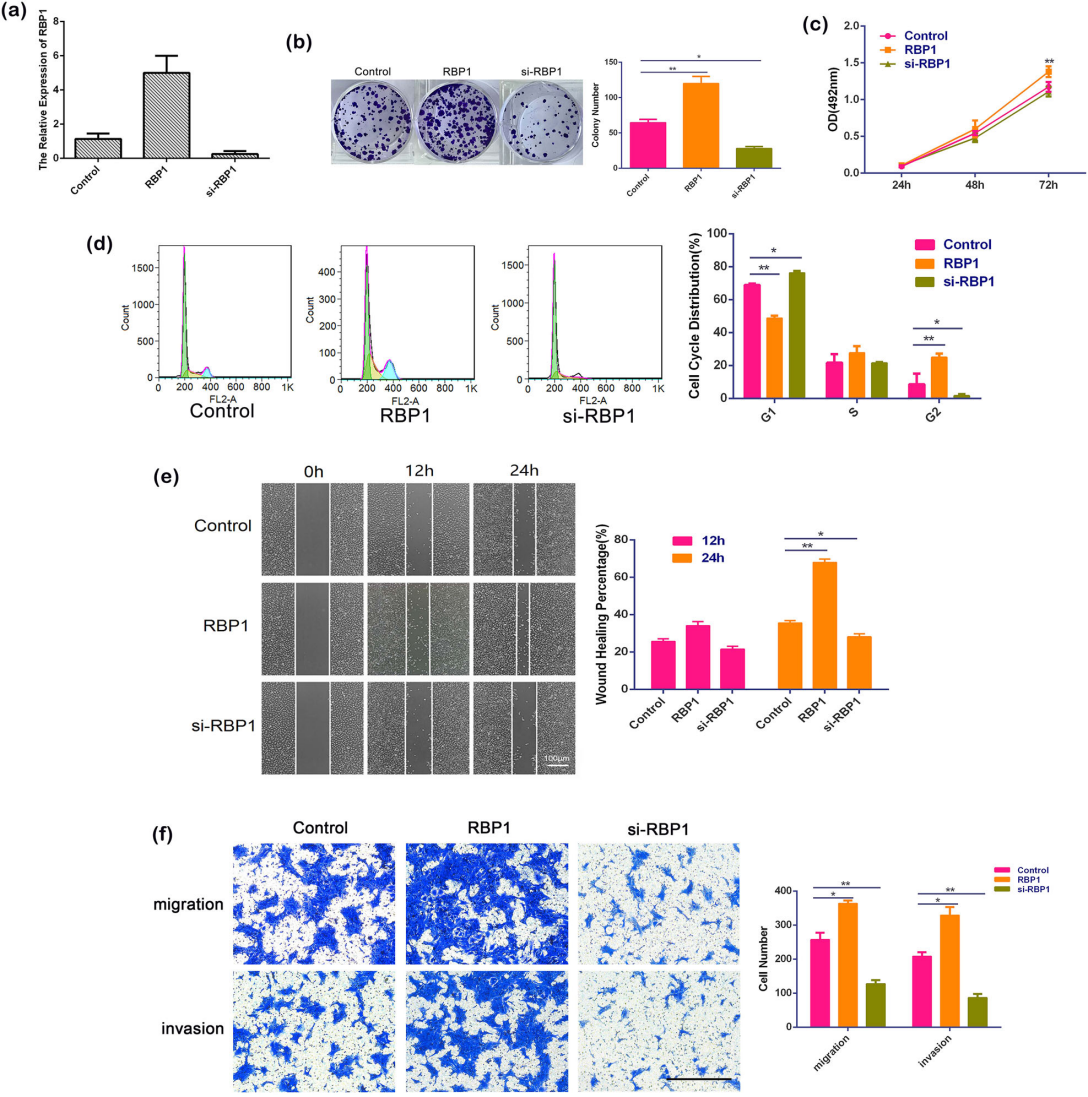
The effects of RBP1 on OSCC growth, migration, and invasion in vitro. **a** Effective overexpression and elimination of RBP1 mRNA in SCC15 cells were achieved, as determined by qRT-PCR analysis of total RNA preparations of these cells. **b** After transfection with control plasmid, RBP1 plasmid, or si-RBP1, the image of 1000 cells were severally plated in a six-well plate for 12 days; the average colony number of per well. **c** At 24, 48, and 72 h after transfection with control plasmid, RBP1 plasmid, or si-RBP1, cell growth was examined by the MTT assay. **d** The relative proportion of SCC15 cell cycle, after transfection with control plasmid, RBP1 plasmid, or si-RBP1. **e** The view of wound-healing migration assay; the average rate of SCC15 cells migration at 0, 12, and 24 h after treatment with control plasmid, RBP1 plasmid, or si-RBP1. Scale bar: 100 μm. **f** The results of the transwell assay of SCC15 cells at 24 h after treatment with control plasmid, RBP1 plasmid, or si-RBP1; the relative ratio of migrated and invasive cells per field was shown. All data were shown as mean ± SD. Scale bar: 100 μm; *P < 0.05, **P < 0.01.

Our clinical pathological data suggested that RBP1 overexpression might play a role in OSCC progression. Hence, we conducted in vitro functional experiments using SCC15 cells to further investigate its role. RBP1-overexpressed SCC15 cells were established by introducing RBP1 plasmid and RBP1 knockout the cells with RBP1 gene silencing mediated by small interfering RNA (si-RBP1). qRT-PCR analysis revealed that there was an effective overexpression or elimination of RBP1 mRNA in SCC15 cells, when the cells were introduced with RBP1 overexpression plasmid or si-RBP1 (Fig. 2a).

Next, we analyzed the cell growth using colony formation analysis, MTT, and cell cycle assay. Wound-healing migration assay, transwell cell migration, and invasion assays were used to evaluate the migrative and invasive capacities of OSCC cells. Colony formation analysis revealed a significantly higher colony forming efficiency in RBP1-overexpressed SCC15 cells compared with the untreated SCC15 cells (119.22 ± 10.50 vs. 64.67 ± 4.51, *P* < 0.01). While those treated with si-RBP1 showed a significantly lower colony forming efficiency compared with untreated cells (28.00 ± 2.65 vs. 64.67 ± 4.51, *P* < 0.05; Fig. 2b). Similarly, MTT assay demonstrated that cell proliferative ability in RBP1-overexpressed cells was promoted compared to the untreated cells (Fig. 2c, *P* < 0.01). Furthermore, flow cytometric analysis of cell cycle distribution showed that RBP1 overexpression accelerated cell cycle at G1 phase and blocked G2 phase progression (Fig. 2d, *P* < 0.01), whereas the distribution in si-RBP1 cells was opposite (Fig. 2d, *P* < 0.05). As revealed by the wound-healing migration assay at 24 h, RBP1-overexpressed cells exhibited greater migration ability that was evidenced by the narrowed healing borders compared to the untreated cells (Fig. 2e, *P* < 0.01). This phenomenon was significantly reversed by si-RBP1 (Fig. 2e, *P* < 0.05). However, no significant change of the cell migration was observed at 12 h (Fig. 2e). Consistently, the transwell assay revealed that the number of the migrated cells (363.67 ± 8.74 vs. 257.00 ± 20.51; Fig. 2f, *P* < 0.05) that were determined by counting the cells on the lower side of the filter and invasive cells (328.67 ± 24.34 vs. 208.33 ± 11.68; Fig. 2f, *P* < 0.05) that were determined by counting the cells through extracellular matrix, were both significantly elevated in RBP1-overexpressed culture. Whereas the numbers of migrated cells (127.33 ± 10.69 vs. 257.00 ± 20.51; Fig. 2f, *P* < 0.01) and invasive cells (86.67 ± 10.69 vs. 208.33 ± 11.68; Fig. 2f, *P* < 0.01) were significantly reduced compared to the untreated cells in presence of si-RBP1. Taken together, these results from in vitro indicated that RBP1 overexpression could enhance the cell growth, as well as migratory and invasive capabilities of SCC15 cells.

### Downregulation of RBP1 suppressed the tumorigenic ability in vivo

**Fig. 3:**
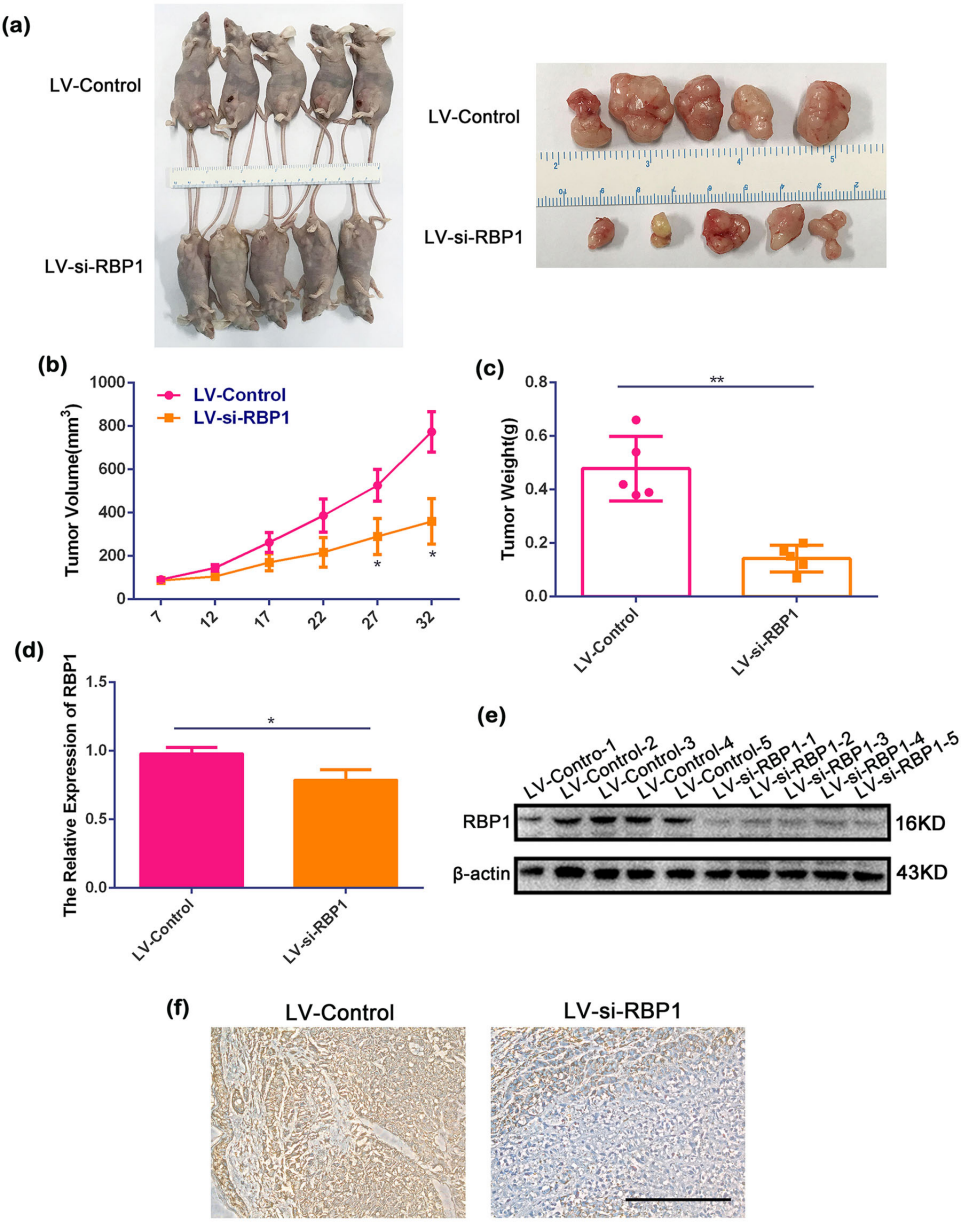
LV-si-RBP1 suppressed tumor growth in vivo. **a** Representative graph showing tumor growth 32 days after subcutaneously implanted OSCC cells. **b**, **c** Calculated tumor volume and weight in LV-si-RBP1 and LV-control groups. **d**, **e** Expression of RBP1 as measured by qRT-PCR and western blot in LV-si-RBP1 and LV-control groups. **f** RBP1 expression was measured by immunohistochemistry staining in LV-si-RBP1 and LV-control groups. Scale bar: 100 μm; *P < 0.05, **P < 0.01.

To verify whether RBP1 plays a role in tumor generation and growth in vivo model, SCC15 cells transfected with si-RBP1 lentivirus (LV-si-RBP1) were injected subcutaneously into the nude mice. The cells transfected with control lentivirus (LV-control) were injected simultaneously as a control. A total of ten mice were injected with two cell subpopulations, LV-si-RBP1 and LV-control (Fig. 3a). According to our observation, the tumors were formed from day 7 after injection. The tumors exhibited the appearance of irregular exophytic, circumscribed, horny masses, and a cauliflower-like surface from day 22 onward. The tumors were collected on day 32 and a reduced tumor volume was noticed in the mice injected with LV-si-RBP1 cells compared to the control groups (Fig. 3a).

Tumor growth curves showed a slower tumor growth potential that reached significance on days 27 and 32 after injection compared with control groups (Fig. 3b, *P* < 0.05). The measurement for tumor weight displayed a reduced tumor weight (Fig. 3c) in the mice injected with LV-si-RBP1 cells in contrast to those injected with LV-control cells (Fig. 3c, *P* < 0.01). The tumors were collected and further analyzed using qRT-PCR, IHC, and western blot analysis. In the LV-si-RBP1 group, gene expression analysis demonstrated that RBP1 was decreased in the LV-si-RBP1 group compared with the LV-control group (Fig. 3d, *P* < 0.05). Western blot analysis and IHC showed that RPB1 was downregulated in the LV-si-RBP1 group vs. control group (Fig. 3e, f). Overall, these results indicated that overexpression of RBP1 could increase in vivo tumorigenic ability.

### RBP1-induced autophagy in OSCC cells

**Fig. 4:**
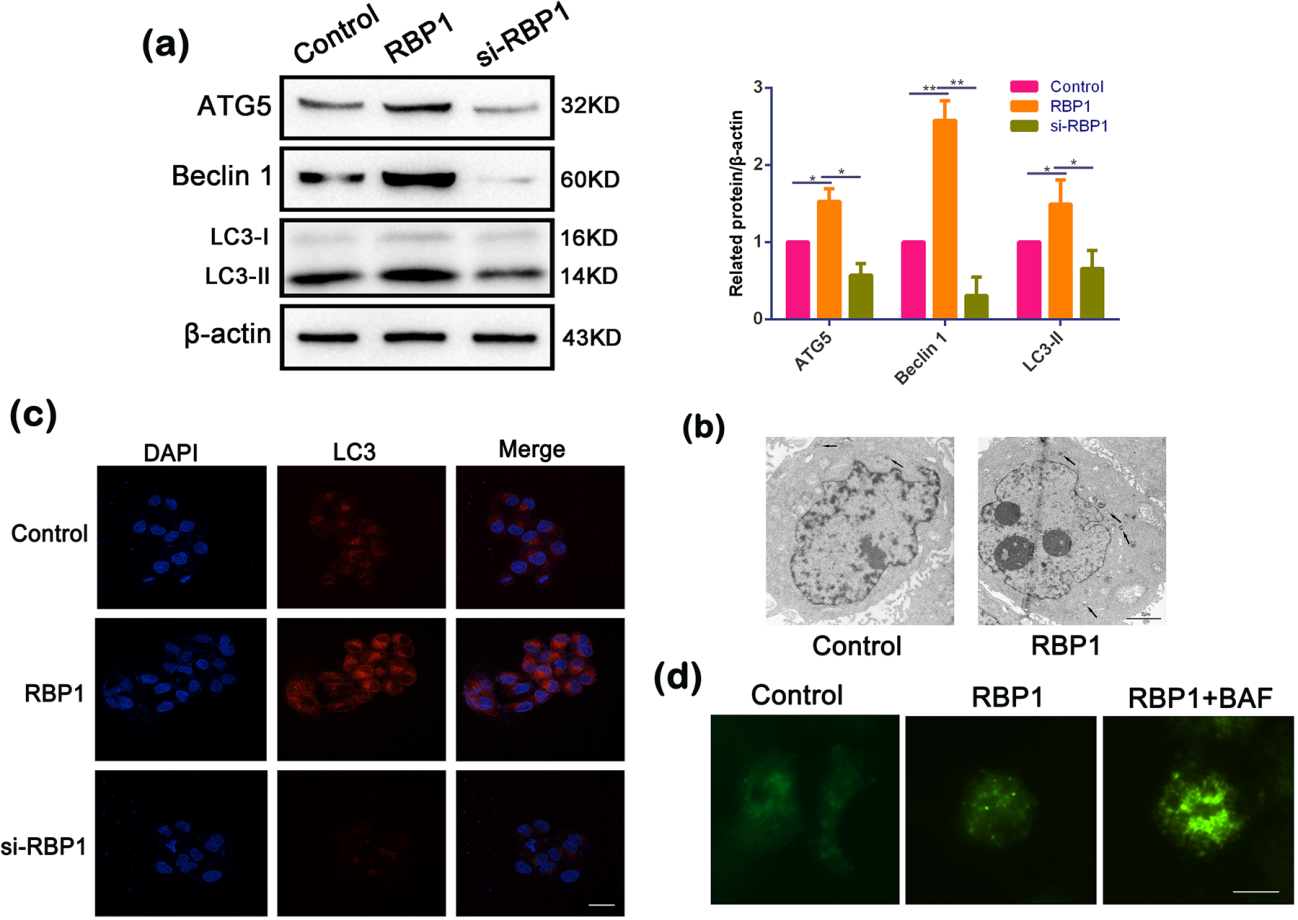
RBP1-induced autophagy and enhanced autophagic flux in OSCC cells. **a** Western blot analysis showing the expression levels of ATG5, BECN1, LC3-II, and β- actin in the cell transfected with control, RBP1 plasmid, or si-RBP1. Quantification of the amount of ATG5, BECN1, and LC3-II to the amount of β- actin for each of the indicated sample. **b** Electron microscopic examination showing autophagic vacuoles (arrows) in the cytoplasm in the cell transfected with control and RBP1 plasmid. Scale bar: 2 μm. **c** Immunofluorescent staining showing the number of LC3 dot in the cell transfected with control, RBP1 plasmid, or si-RBP1. Cells were stained by indirect immunofluorescence using anti-LC3 antibody, and the number of LC3 dot per cell was quantified. Scale bar: 20 μm. **d** RBP1 overexpression enhanced autophagic flux. The number of GFP-LC3 dot in the RBP1-overexpressed cells and control cells treated bafilomycin A1 (BAF). Cells were transfected with GFP-LC3 adenovirus. 24 h after transfection, cells were treated with BAF, then GFP-LC3 dot was examined by fluorescence microscopy. Scale bar: 10 μm; *P < 0.05, **P < 0.01.

Autophagy is a ubiquitous cellular pathway that linked to protein turnover^29^ and involved in tumor development in several cancers^30,31^. To further determine whether RBP1 regulates autophagy, we examined the expression levels of autophagy-associated proteins, including Beclin 1, ATG5, and determined the lipidation of endogenous LC3-I to yield LC3-II that marks the occurrence of an autophagy process. A significant increase was detected in the expression of Beclin 1, ATG5 along with LC3-II/LC3-I in RBP1-overexpressed cells, while a significant decrease was observed in si-RBP1 cells compared to untreated cells (Fig. 4a). We also found that autophagic vacuoles were augmented in RBP1-overexpressed cells by electron microscopy (Fig. 4b). To detect the LC3 distribution, we then performed immunofluorescent staining and found that the stained dots of LC3 was considerably augmented in the RBP1-overexpressed cells, while these were reduced in the cells transfected with si-RBP1 compared to untreated cells (Fig. 4c). These results provided evidence regarding the occurrence of RBP1-induced autophagy in OSCC cells.

LC3-II accumulation indicates increased autophagosome formation in the upstream or impaired autophagy–lysosome fusion in the downstream. In order to distinguish these two different processes, the LC3-II levels were detected in the presence of bafilomycin A1 (BAF) in order to distinguish between these two different processes. BAF is an autophagy inhibitor that prevents downstream autophagy–lysosome fusion. The expression of lentivirus transfected with RBP1-overexpressed cells was fused with LC3 for 24 h, and the number of lentivirus-expressing GFP fused with LC3 (GFP-LC3) points was observed after BAF treatment for 24 h. We found that BAF increased the amount of GFP-LC3 dot induced by RBP1-overexpressed cells (Fig. 4d). Collectively, these results indicated that RBP1 overexpression promoted the occurrence of cell autophagy in OSCC cells.

### Inhibition of autophagy reversed the carcinogenic effects of RBP1 on OSCC cells

**Fig. 5:**
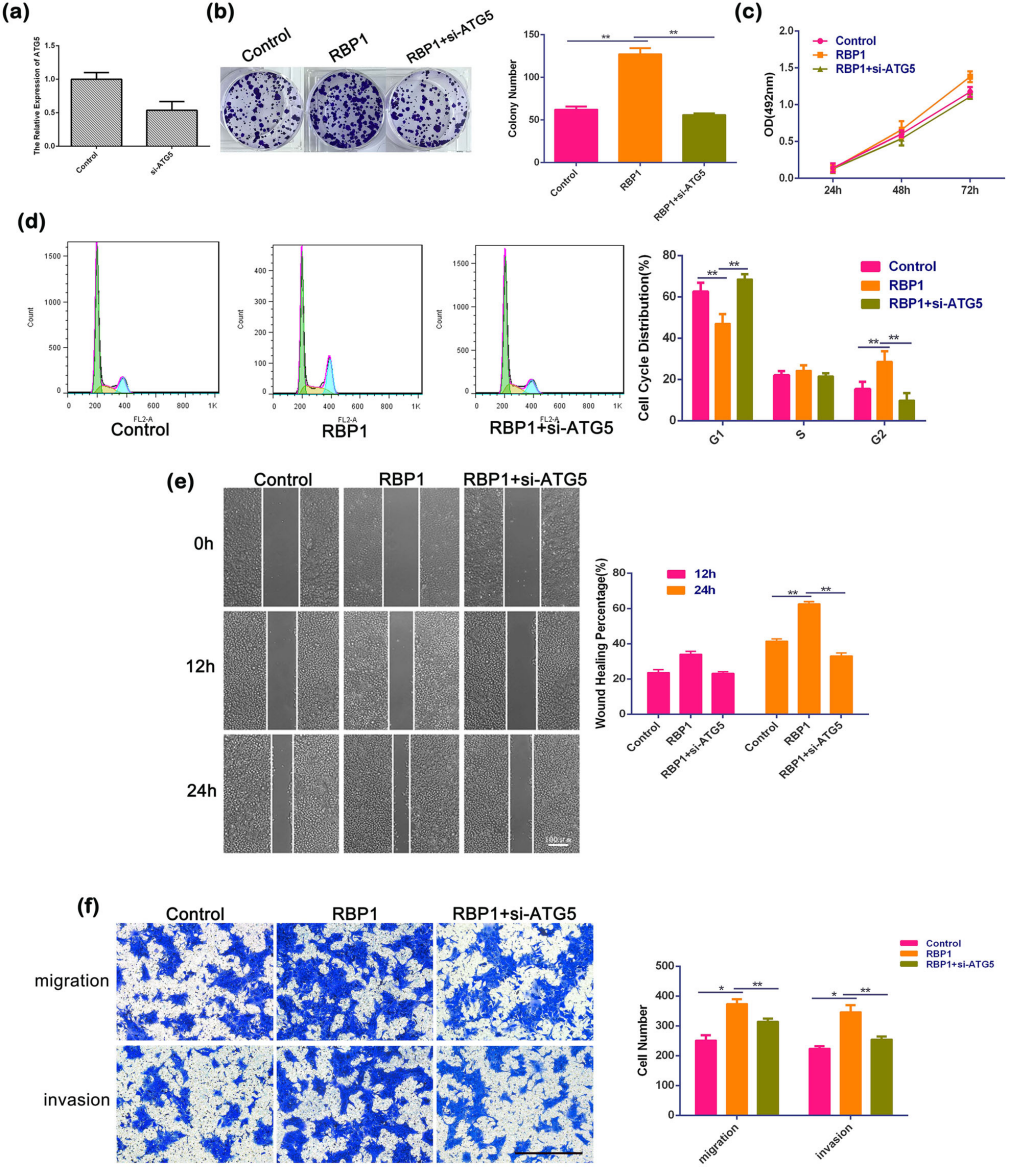
The effects of ATG5 siRNAs on growth, migration, and invasion of RBP1 overexpression OSCC cells in vitro. **a** Effective elimination of ATG5 mRNA in SCC15 cells were achieved, as determined by qRT-PCR analysis of total RNA preparations of these cells. **b** After transfection with control plasmid, RBP1 plasmid, or RBP1 plasmid with si-ATG5, the image of 1000 cells were severally plated in a six-well plate for 12 days; the average colony number of per well. **c** At 24, 48, and 72 h after transfection with control plasmid, RBP1 plasmid, or RBP1 plasmid with si-ATG5, cell growth was examined by the MTT assay. **d** The relative proportion of SCC15 cell cycle, after transfection with control plasmid, RBP1 plasmid, or RBP1 plasmid with si-ATG5. **e** The view of wound-healing migration assay; the average rate of SCC15 cells migration at 0, 12, and 24 h after treatment with control plasmid, RBP1 plasmid, or RBP1 plasmid with si-ATG5. Scale bar: 100 μm. **f** The results of the transwell assay of SCC15 cells at 24 h after treatment with control plasmid, RBP1 plasmid, or RBP1 plasmid with si-ATG5; the relative ratio of migrated and invasive cells per field was shown. Scale bar: 100 μm; *P < 0.05, **P < 0.01.

In order to determine whether regulation of autophagy by RBP1 is involved in cellular growth, migration, and invasion of OSCC cells, siRNA against ATG5 (si-ATG5) was applied to inhibit autophagy in the RBP1-overexpressed cells. Gene expression analysis revealed that there was effective elimination of ATG5 mRNA in SCC15 cells when the cells were transfected with si-ATG5 (Fig. 5a). Results of colony formation assay showed that RBP1 significantly increased the capability of colony formation (127.33 ± 7.02 vs. 62.33 ± 3.51; Fig. 5b, *P* < 0.01) and proliferative capabilities of the cells (56.00 ± 1.73 vs. 127.33 ± 7.02; Fig. 5b, *P* < 0.01) in si-ATG5, with RBP1-overexpressed cells relative to RBP1 cells, respectively. A similar finding was observed in MTT assay (Fig. 5c). Flow cytometric analysis showed that si-ATG5 blocked cell cycle at G1 phase and accelerated G2 phase progression in the cells transfected with RBP1 overexpression (Fig. 5d, *P* < 0.01). Moreover, wound-healing assay showed that si-ATG5 cells had less migration ability that was evidenced by the wider healing borders compared to the control RBP1 cells (Fig. 5e, *P* < 0.01). Consistently, the transwell assay revealed that the number of the migratory cells (314.67 ± 10.26 vs. 374.00 ± 15.52; Fig. 5f, *P* < 0.01) and invasive cells (255.00 ± 9.85 vs. 346.33 ± 23.03; Fig. 5f, *P* < 0.01) were significantly decreased in RBP1-overexpressed cells transfected with si-ATG5 compared with those in RBP1 culture, respectively. These findings suggested that RBP1 promoted cell growth, migration, and invasion in OSCC cells *via* activation of autophagy.

### RBP1 interacted with CKAP4

**Fig. 6:**
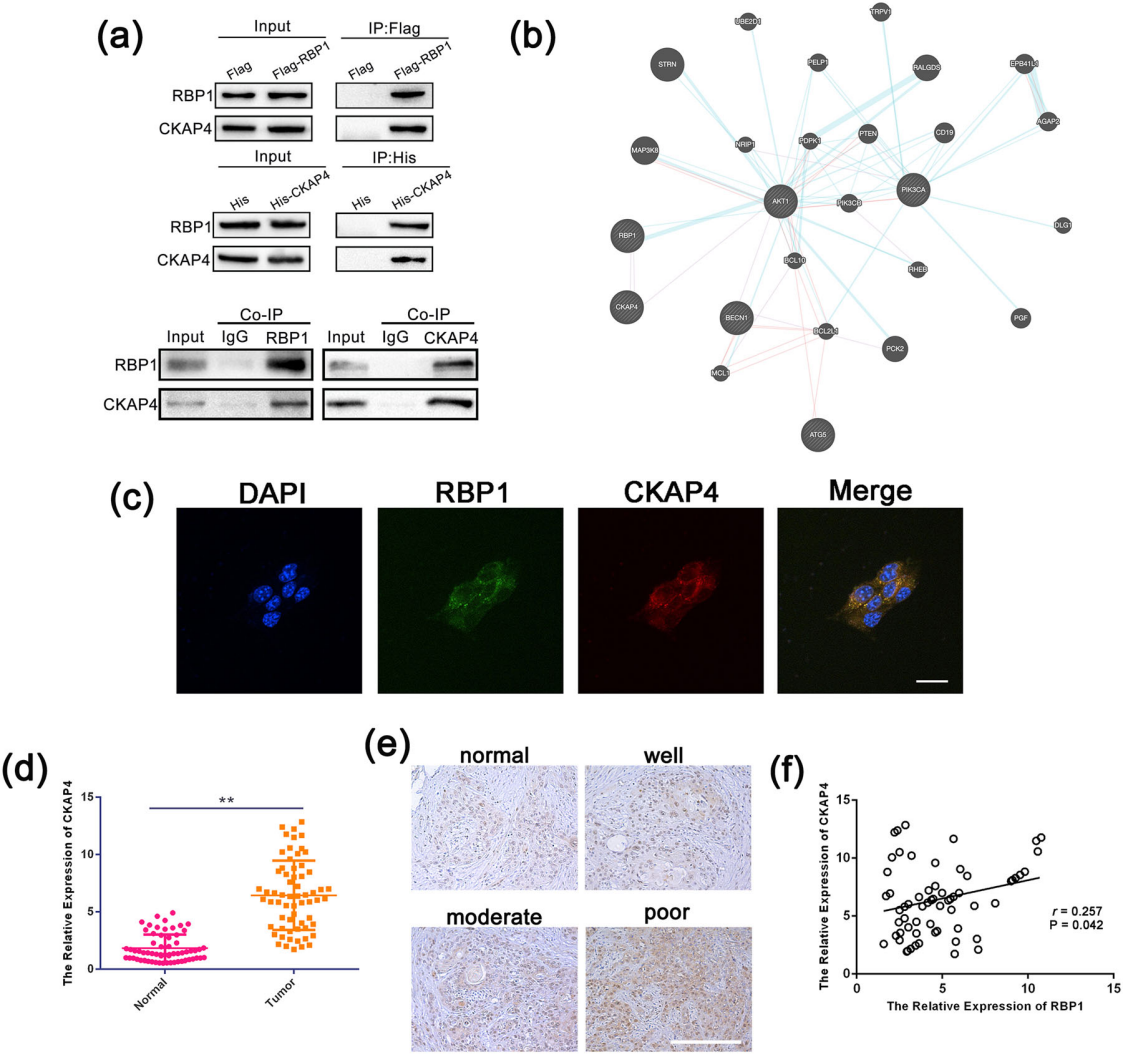
RBP1 interacted with CKAP4. **a** Interaction between RBP1 and CKAP4 by Co-IP analyses in SCC15 cells. **b** The co-expressed network of RBP1 and CKAP4 in tumor tissues. **c** Co-localization analysis RBP1 and CKAP4 in SCC15 cells co-transfected with RBP1 and CKAP4. **d** The expression of CKAP4 in 63 pairs of OSCC tissues and normal tissues were analyzed by qRT-PCR. **e** IHC was used to analyze the expression of CKAP4 in 63 pairs of OSCC tissues and normal tissues. Scale bar: 100 μm. **f** Correlation analysis of the expression level of RBP1 and CKAP4; **P < 0.01.

Identifying the partners of a protein and protein–protein interactions may lead to understand the underlying molecular mechanisms. In order to characterize protein–protein interactions, we carried out (co-immunoprecipitation (Co-IP)) followed by MS to explore the RBP1 interacting proteins. We performed Co-IP followed by MS using the lysate of SCC15 cells transfected with RBP1 or CKAP4. CKAP4/P63 was identified as one of the potential RBP1 interacting proteins among a panel of the identified proteins. The correlation between RBP1 and CKAP4 (Fig. 6a) was further confirmed by the Co-IP assay. We then performed bioinformatics (https://genemania.org/) and found an association between RBP1 and CKAP4 (Fig. 6b). To further confirm this interaction, we next examined the localization in SCC15 cells co-transfected with RBP1 and CKAP4, which were performed by fluorescence immunocytochemistry. Results obtained from these experiments demonstrated that the co-localization of RBP1 and CKAP4 in cytoplasm (Fig. 6c).

Next, we tested the expression of CKAP4 in 63 pairs of OSCC tissues and normal tissues. Upregulation of CKAP4 was seen in OSCC tissues compared to the adjacent nontumor tissues (Fig. 6d, e). Meanwhile, we also did qRT-PCR to examine the expression of CKAP4 in OSCC cell lines and HOK cells. The results were consistent with that of clinical tissues (Supplementary Fig. 1). In addition, Pearson correlation analysis exhibited a positive correlation between the expression of RBP1 and CKAP4 in OSCC tissues (Fig. 6f). These results indicated the significant correlation between RBP1 and CKAP4 in OSCC.

### CKAP4-induced autophagy in OSCC cells

**Fig. 7:**
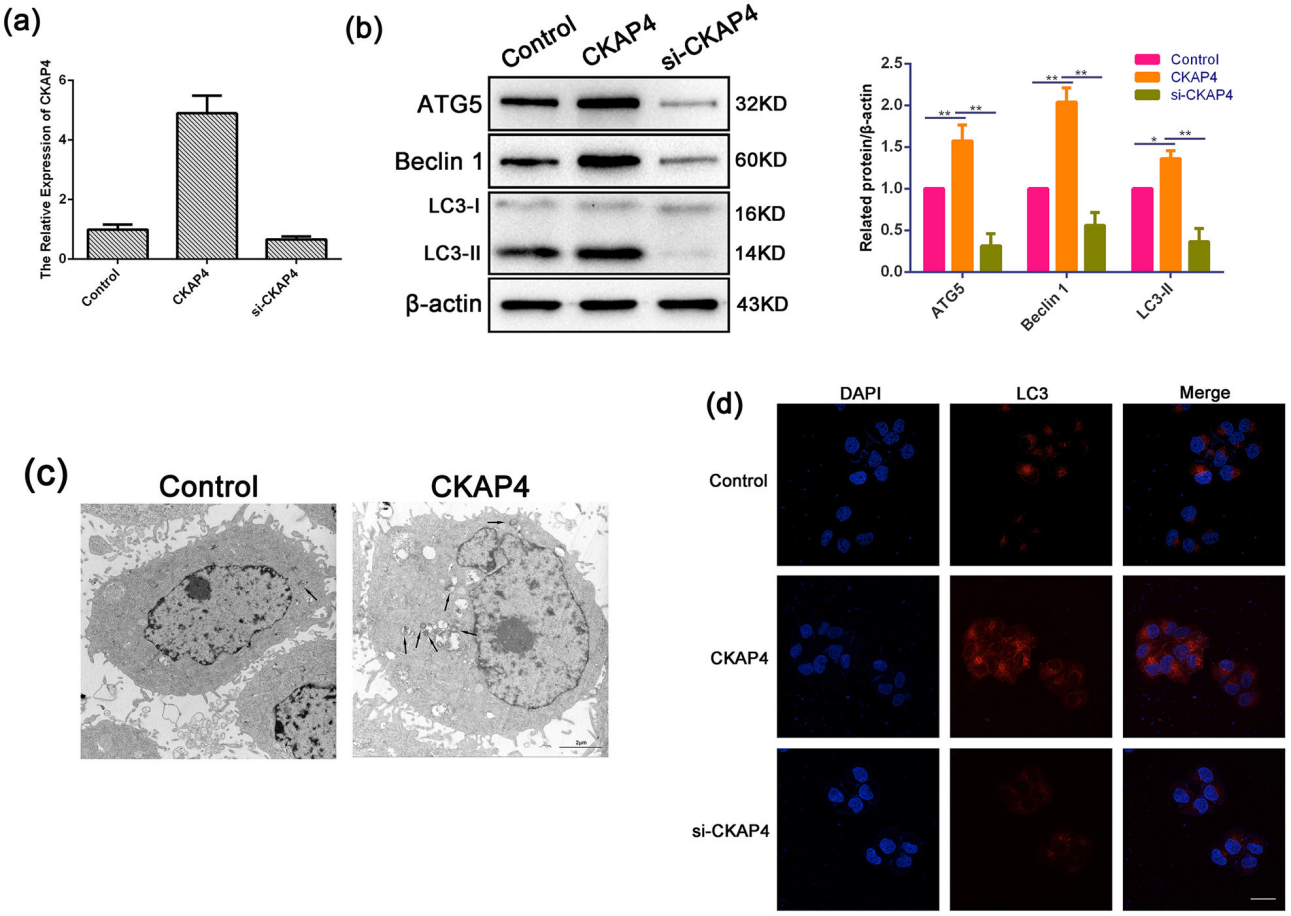
CKAP4-induced autophagy in OSCC cells. **a** Effective overexpression and elimination of CKAP4 mRNA in SCC15 cells were achieved, as determined by qRT-PCR analysis of total RNA preparations of these cells. **b** Western blot analysis showing the expression levels of ATG5, BECN1, LC3-II, and β-actin in the cells transfected with control, CKAP4 plasmid, or si-CKAP4. Quantification of the amount of ATG5, BECN1, and LC3-II to the amount of β-actin for each of the indicated sample. **c** Electron microscopic examination showing autophagic vacuoles (arrows) in the cytoplasm in the cell transfected with control and CKAP4 plasmid. Scale bar: 2 μm. **d** Immunofluorescent staining showing the number of LC3 dot in the cell transfected with control, CKAP4 plasmid, or si-CKAP4. Cells were stained by indirect immunofluorescence using anti-LC3 antibody, and the number of LC3 dot per cell was quantified. Scale bar: 20 μm; *P < 0.05, **P < 0.01.

To further explore the role of CKAP4 in regulating autophagy. Basing on Fig. 6b, a potential relationship between CKAP4 and autophagy protein Beclin 1 was observed, which is essential at the initial stages of the autophagosome formation^32^. We hypothesized that CKAP4 regulated autophagy in OSCC. In order to test this hypothesis, we examined the levels of Beclin 1, ATG5, and determined the lipidation of endogenous LC3-I to yield LC3-II in the SCC15 cell lines with either CKAP4 overexpressed or gene silencing (siRNA of CKAP4 (si-CKAP4)). qRT-PCR analysis demonstrated that an effective overexpression or elimination of CKAP4 mRNA in SCC15 cells was achieved (Fig. 7a). As shown in Fig. 7b, we also detected a significantly increased expression of Beclin 1, ATG5, and LC3-II/LC3-I in the CKAP4-overexpressed cells, while a significant reduction in si-CKAP4 cells compared to control cells. Furthermore, we found that the amounts of autophagic vacuoles were strikingly augmented in CKAP4-overexpressed cells compared with control cells using electron microscopy (Fig. 7c). Immunofluorescent staining demonstrated the significantly increased LC3 expression with an appearance of dot-staining pattern in the CKAP4-overexpressed cells, while these were significantly reduced in the cells transfected with si-CKAP4 (Fig. 7d). These results suggested the occurrence of CKAP4-induced autophagy in OSCC cells.

### RBP1-induced autophagy enhanced cell growth, migration, and invasion of OSCC cells via CKAP4 axis

**Fig. 8:**
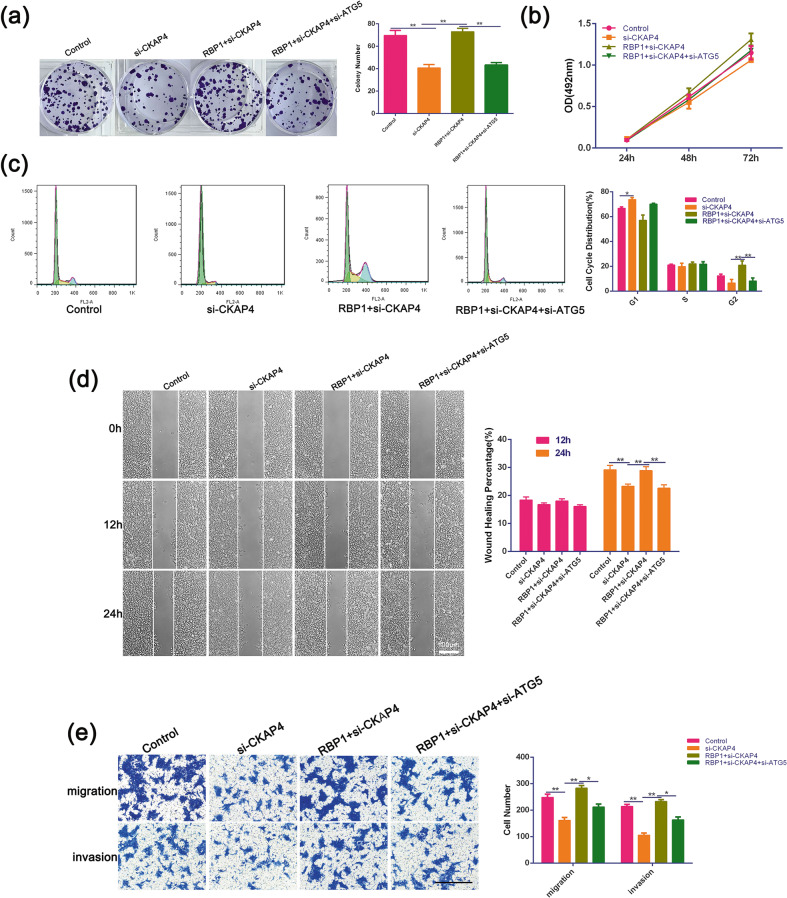
The effects of ATG5 siRNAs on growth, migration, and invasion of RBP1 and si-CKAP4 OSCC cells in vitro. **a** After transfection with control vector, si-CKAP4, RBP1 with si-CKAP4, or RBP1 + si-CKAP4 + si-ATG5, the image of 1000 cells were severally plated in a six-well plate for 12 days; the average colony number of per well. **b** At 24, 48, and 72 h after transfection with control, si-CKAP4, RBP1 with si-CKAP4, or RBP1 + si-CKAP4 si-ATG5, cell growth was examined by the MTT assay. **c** The relative proportion of SCC15 cell cycle, after transfection with control plasmid, si-CKAP4, RBP1 with si-CKAP4, or RBP1 + si-CKAP4 + si-ATG5. **d** The view of wound-healing migration assay; the average rate of SCC15 cells migration at 0, 12, and 24 h after treatment with control plasmid, si-CKAP4, RBP1 with si-CKAP4, or RBP1 + si-CKAP4 + si-ATG5. Scale bar: 100 μm. **e** The results of the transwell assay of SCC15 cells at 24 h after treatment with control plasmid, si-CKAP4, RBP1 with si-CKAP4, or RBP1 + si-CKAP4 + si-ATG5; the relative ratio of migrated and invasive cells per field was shown. Scale bar: 100 μm; *P < 0.05, **P < 0.01.

To explore the effects of CKAP4 together with induced autophagy on cellular growth, migration, and invasion, we applied si-ATG5 to inhibit autophagy in the cells co-transfected with RBP1 and si-CKAP4. Silencing of CKAP4 considerably inhibited colony forming efficiency (Fig. 8a), the cell growth (Fig. 8b), cell migration (Fig. 8d, e), and invasion (Fig. 8e) in the assays for colony formation, MTT, wound healing and transwell, respectively. Overexpression of RBP1 further reversed the effects of CKAP4 depletion, while double-knockdown of CKAP4 and ATG5 reduced the RBP1-dependent increase in colony formation efficiency (Fig. 8a), cell migration (Fig. 8d, e), and invasion (Fig. 8e) to the level of control cells. Moreover, si-CKAP4 blocked cell cycle at G1 phase, and accelerated G2 progression compared to control and RBP1 and si-CKAP4 co-transfected groups (Fig. 8c). Taken together, the above results suggested that RBP1-induced autophagy enabled to enhanced cell growth, migration, and invasion in OSCC cells via CKAP4 axis.

## Discussion

There is increasing evidence pointing toward the significance of RBP1 in tumor development and progression^8,15^. In this study, we have provided evidence for RBP1 as a biomarker in OSCC, and its novel role as an inducer of autophagy that further promotes in vitro and in vivo tumor cell growth via interaction with CKAP4 (Supplementary Fig. 2).

RBP1 is a small cytosolic protein of 15 KDa (ref. ^13^) and functions as a chaperone protein for regulation of retinol uptake, and its subsequent esterification and its bioavailability. RBP1 binds to retinol and interacts with enzymatic esterification of retinol with long chain fatty acids and hydrolysis of retinol esters to retinol^33^. In addition, RBP1 has been reported to affect the metabolism of retinoic acid by reducing the transport of retinol, blocking the formation of retinol esters and RARs activity, leading to cell differentiation and tumor progression^34^, indicating that RBP1 might be crucial in the development of cancers.

In our study, OSCC and normal tissues were comparatively profiled with iTRAQ-based MS, which can identify any type of protein, and simultaneously label eight samples to quantify the differential proteins in multiple samples. However, till date there is very limited research on the utilization of iTRAQ to study OSCC DEPs. Our previous study has identified a tumor suppressor PIP (prolactin-induced protein) and explored its potential benefit as a new tumor biomarker of OSCC (ref. ^28^). In this present study, RBP1 was chosen as a candidate in the current study using iTRAQ. Previous studies reported that RBP1 was increased in HCT116-derived radiation-resistant cells of rectal cancer^35^. In addition, upregulated expression of CRBP-1 was associated with a poor prognosis in tongue squamous cell carcinoma patients. Consistent with above results, our data revealed a significant upregulation of RBP1 in 63 OSCC tissues, as well as in OSCC cell lines. Furthermore, results from functional experiments demonstrated that upregulation/downregulation of RBP1 promoted/inhibited cell growth, migration, and invasion of OSCC cells in vitro, RBP1 knockout repressed the tumor growth in vivo. These findings indicated that upregulation of RBP1 was associated with tumorigenesis and progression in OSCC, which could potentially serve as a diagnostic and prognostic biomarker of OSCC.

Many studies have reported that abnormal regulation of autophagy is closely related to the occurrence and development of tumors^30,36^. Although under certain circumstances autophagy suppresses tumorigenesis, it is mostly a facilitator of tumorigenesis^31^. For example, Wu et al.^37^ reported that TNF receptor-associated factor 6 inhibited epithelial–mesenchymal transition and colorectal cancer metastasis *via* autophagic degradation machinery. FOXM1 expression was upregulated in the docetaxel resistant prostate cancer cell lines, downregulation of ATG7, Beclin 1, or cotreatment with chloroquine partly restored sensitivity to docetaxel in the FOXM1-overexpressing prostate cancer cells^38^. Similarly, in the present study, we demonstrated, for the first time, that upregulation of RBP1 enabled an increase in the expression levels of Beclin 1, ATG5, and LC3-II, as well as the autophagy and the autophagic flux of OSCC cells. In contrast, downregulation of RBP1 inhibited the effects of autophagy. Furthermore, silencing of ATG5 rescued RBP1 overexpression-induced malignant biological behaviors, including growth, migration, and invasion of OSCC cells. These findings suggested that inhibition of RBP1-induced autophagy decreased tumor growth and progression of OSCC.

CKAP4 functions as a Dkk1 receptor, which was originally discovered as a protein that is mainly localized to the ER (ref. ^17^). There is emerging evidence, which has indicated that dysregulation of Dkk1 was implicated in the pathogenesis of a variety of cancers^23,39,40^. Previous study demonstrated that activation of the Dkk1-CKAP4 pathway was associated with a poor prognosis of cancer^40^. CKAP4 promoted cancer cell growth via the PI3K/AKT signaling pathway in several cancer cell lines, including pancreatic ductal adenocarcinoma^41^ and pancreatic cancer^39^. Consistently, our data revealed the upregulation of CKAP4 in OSCC tissues and cell lines. Based on bioinformatics analysis and cell functional experiments, we found that RBP1 directly interacted with CKAP4, upregulation of RBP1 reversed the antitumor effects of si-CKAP4 on autophagy and malignant biological behaviors of OSCC. In addition, the network between RBP1, CKAP4, PI3K, AKT, ATG5, and Beclin 1 was predicted, Beclin 1 functions as an allosteric modulator of PI3K complex, which is essential at the initial stages of the autophagosome formation^32,42^. ATG5 is also indispensable for autophagic vesicle formation^43^. Our study demonstrated that ATG5 silencing significantly rescued the carcinogenic effects of RBP1 on activation of autophagy and malignant biological behaviors of OSCC. These results indicated that RBP1 activated CKAP4 to promote the malignant progression of OSCC through autophagy activation.

In conclusion, results from our study have demonstrated that RBP1 could be a potential novel biomarker and antitumor target in OSCC. Furthermore, we elucidated the underlying molecular mechanisms involved by providing evidence that RBP1 activated autophagy through the modulation of CKAP4. Intriguingly, we further demonstrated that CKAP4 and autophagy/ATG5 knockdown inhibited growth, migration and invasion of OSCC cells. Therefore, inhibition of RPB1–CKAP4-induced autophagy can be further exploited as a potential therapeutically target for in the treatment of OSCC.

## Materials and methods

### Specimens collection and cell culture

Clinical samples were collected from patients diagnosed with OSCC from January 2013 to December 2018 at Department of Oral and Maxillofacial Surgery, the Affiliated Hospital of Qingdao University. Samples were taken from the primary OSCC tumor surgically removed and from paired samples of adjacent normal tissue. No patients received chemotherapy or radiotherapy prior to surgery. Ethical approval was approved by local Ethics Committee and all patients signed informed consent forms of tissue samples used in this study. SCC15, SCC25, SCC9, CAL27, and HOK cell lines were purchased and identified from the Chinese Academy of Medical Sciences (Beijing, China).

### Cell culture and transfection

High-glucose DMEM medium (HyClone, USA) with the addition of 10% fetal bovine serum (FBS; HyClone, USA) and 1% penicillin/streptomycin solution (Solarbio, China) was used for maintaining the cells. Flasks containing the cells were cultured at 37 °C and supplied with 5% CO_2_. Logarithmic phase cells were used in all experiments. RBP1 and CKAP4 plasmids were generated by Vigene Biosciences (Shandong, China). The si-RBP1, si-ATG5, and si-CKAP4 were designed by GenePharma (Shanghai, China). The cells were transfected using Lipofectamine 3000 (Thermo Fisher Scientific, USA).

### iTRAQ

The labeled peptides were mixed and classified by Agilent 1260 infinity II HPLC system (Agilent, Beijing, China). Buffer solution A was 10 mM HCOONH_4_, 5% CAN, pH 10.0. Buffer solution B was 10 mM HCOONH_4_, 85% CAN, pH 10.0. The column was then balanced using liquid A. Samples were added to the column for separation with A manual sampler, and separated at A flow rate of 1 ml/min. Absorbance value (214 nm) was monitored during elution. The elution components were collected at an interval of 1 min, with a total of 36 components. The samples were then lyophilized and then mixed with 0.1% formic acid (FA).

### LC–MS/MS

Each sample was separated using the Easy nLC system (Thermo Fisher Scientific, CA). Buffer A was 0.1% FA solution, buffer B was 0.1% acetonitrile solution (acetonitrile was 80%). The chromatographic column was balanced with 100% buffer A and the samples were added to the analytical column with an automatic sampler, and separated at 300 nl/min. After chromatographic separation, the samples were analyzed by Q-exactive mass spectrometer (120 min; Thermo Fisher Scientific, CA).

### Quantitative real-time PCR

The total RNA was separated by using TRIzol reagent (Invitrogen, USA) following the instructions provided by the manufacturer. A Prime Script RT reagent kit (Takara, Japan) was used to make reverse transcription according to the manufacturer’s instruction. SYBR Prime Script RT-PCR kit (Takara, Japan) was used to carry out qRT-PCR by following the manufacturer’s protocol. All mRNA expression levels were calculated by using the 2^−ΔΔCt^ method and were normalized to β-actin expression.

### Cell proliferation, cell cycle, and colony formation assay

Cells were seeded into 96-well plates for 24, 48, and 72 measured using MTT assay for cell proliferation assay. They were fixed with 70% ethanol and stained with propidium iodide. Cell cycle was determined using flow cytometry (BD Biosciences, USA). In colony formation assay, 1000 cells per well were transplanted into six-well plates and cultured for 12 days. Crystal violet staining was used for scoring viable colonies.

### Wound-healing and transwell assays

For wound-healing assay, cells were transplanted into six-well plates. When cellular density reached nearly 100%, the cell monolayer was “wounded” in a cross-shaped pattern with a 200 μl micropipette tip followed with washing by phosphate-buffered saline (PBS). Then the hungry cells were cultured in the medium without FBS. Images of the wound were taken under a camera (Nikon, Kobe, Japan) at 0, 12, and 24 h respectively. The scratch areas were measured using ImageJ software (National Institutes of Health, USA).

For transwell assay, the chambers (8-μm pore, Corning Costar, USA) without or with matrix gel (100 μg/ml, BD Biosciences, CA) were utilized to assess the abilities of migration and invasion. For migration assay, the cells were inoculated into the upper compartment and in an FBS-free environment. The lower compartment was full of the medium with 10% FBS. For invasion assay, the matrix gel was precoated on the upper compartment. After 24 h, five fields of view were randomly selected and counted under an optical microscope (Nikon, Kobe, Japan).

### IHC staining

IHC was performed on paraffin-embedded OSCC tissues and adjacent tissues (*n* = 63). It was conducted with 1:200 rabbit anti-RBP1 affinity purification (Proteintech, cat. no. 22683-1-AP) or 1:200 rabbit anti-CKAP4 affinity purification (Proteintech, cat. no. 16686-1-AP) as the primary antibody. Following dewaxing and rehydration, the antigen was removed at 10 mM sodium citrate (PH 6.0), 95 °C for 20 min. Then, the sections were blocked by endogenous peroxidases and goat serum for 10 min, respectively, and were incubated overnight at 4 °C with the primary antibodies. Tissue antigens were observed by DAB (Sangon Biotech, China). Finally, counterstaining was conducted with hematoxylin.

### Xenotransplantation

All animal experiments were conducted in compliance with animal protocols approved by the Affiliated Hospital of Qingdao University. They were carried out at the Animal Laboratory of the Affiliated Hospital of Qingdao University. A total of ten female 5-week-old BALB/C nude mice were purchased from the Animal Laboratory of the Affiliated Hospital of Qingdao University were randomly divided into two groups with each group consisting of five animals. The LV-si-RBP1 was constructed by GeneChem (Shanghai, China), and the lentiviral vector (LV-control) as a blank control. A total of 1 × 10^7^ SCC15 cells transfected with LV-si-RBP1 or LV-control were conducted the posterior inguinal subcutaneous injection. Tumor volume (V) was calculated by measuring the width (W) and length (L) with calipers every 5 days for 25 days using V = (L × W^2^)/2. All nude mice were euthanized after 32 days with carbon dioxide and their tumors were collected and weighed.

### Immunofluorescent staining

SCC15 cells were seeded into slides and cultured with DMEM containing 10% FBS for 12 h. Then, cells were fixed with 4% paraformaldehyde and permeabilized with 0.5% Triton X-100 at room temperature for 30 min. They were washed with 0.01 M PBS and blocked with 5% bull serum albumin for 2 h. Subsequently, cells were incubated with primary antibody anti-LC3B (1:200; Cell Signaling Technology, cat. no. 2775) overnight at 4 °C. Following incubation with the secondary antibody Rhodamine (TRITC)-conjugated Goat Anti-Rabbit IgG (1:100; Proteintech, cat. no. SA00007-2) at 37 °C under dark for 1 h, the slides were counterstained with DAPI (Solarbio, China).

### Transmission electron microscopy

Cells were fixed with 2.5% glutaraldehyde in 0.1 M sodium cacodylate buffer, stored at 4 °C overnight, and postfixed with 1% OsO_4_ for 1 h. After staining with 3% uranyl acetate and dehydrated in a graded ethanol, the cells were embedded in epoxy resin. A transmission electron microscope (JEOL, Tokyo, Japan) was used for observing the ultrathin slices.

### Western blot analysis

The cell lysate was collected using the lysis buffer (Beyotime Biotechnology, Shanghai, China). The whole proteins were scattered through SDS–PAGE on 10% gel and were transferred to polyvinylidene difluoride membranes (Provider). Membranes were blocked using 5% fat-free milk in PBS-Tween 20 and incubated with primary antibodies at 4 °C overnight. The primary antibodies were anti-RBP1 (1:1000; Proteintech, cat. no. 22683-1-AP), anti-ATG5 (1:500; Proteintech, cat. no. 10181-2-AP), anti-Beclin 1 (1:1000; Proteintech, cat. no. 11306-1-AP), anti-LC3B (1:1000; Cell Signaling Technology, cat. no. 2775), and anti-β-actin (1:2000; Proteintech, cat. no. 20536-1-AP). Subsequently, the membranes were incubated with secondary antibody Goat Anti-Rabbit IgG (1:10,000; Proteintech, cat. no. 10285-1-AP) for 1 h at room temperature.

### Co-immunoprecipitation

The Co-IP assay was performed using a Co-IP kit (Thermo Scientific, Waltham, MA, USA). Cells were washed with ice-cold PBS and lysed on ice for 30 min, followed by centrifugation at 14,000 × *g* for 15 min. The supernatants were incubated with the indicated antibodies on a rotator overnight at 4 °C. Then, the mixtures were incubated with immobilized protein A/G beads (Thermo Scientific) on a rotator for 2 h at 4 °C. The beads were collected by centrifugation at 3000 × *g* for 2 min and washed three times with IP lysis buffer. The co-immunoprecipitated proteins were eluted from the beads, resolved by SDS buffer and analyzed by western blotting. The following primary antibodies were used: anti-RBP1 (1:250; SCBT, cat. no. sc-81640), anti-CKAP4 (1:1000; Proteintech, cat. no. 16686-1-AP), and anti-IgG (1:500; Abcam, cat. no. ab200699).

### Statistics

All data were presented as the mean ± SD. Statistical analysis was performed using student’s *t*-test to compare two groups. The association between RBP1 and clinicopathological characteristics in OSCC patients was carried out using the chi-square test. Spearman’s correlation was utilized to explore the association between RBP1 and CKAP4 expression. The significance of statistical analysis was clarified by *P < 0.05 and **P < 0.01.

## Supplementary information

Supplemental Table S1

Supplementary Figure Legends

Supplemental Fig. S1

Supplemental Fig. S2
