# Supplementary material for: The RBP1–CKAP4 axis activates oncogenic autophagy and promotes cancer progression in oral squamous cell carcinoma
Source: Cell Death Dis. 2020 Jun 25;11(6):488. doi: 10.1038/s41419-020-2693-8 (PMC7316825; doi:10.1038/s41419-020-2693-8)
Supplement: Supplementary file 1 — Supplemental Table S1 [file 41419_2020_2693_MOESM1_ESM.doc]

| **Variable** | **n** | **Expression level** | | ***P*** |
| --- | --- | --- | --- | --- |
|  |  | **High** | **Low** |  |
| Age |  |  |  | 0.889 |
| <60 years | 29 | 20 | 9 |  |
| ≥60 years | 34 | 24 | 10 |  |
| Gender |  |  |  | 0.459 |
| Male | 32 | 21 | 11 |  |
| Female | 31 | 23 | 8 |  |
| Differentiation |  |  |  | 0.026 |
| High | 33 | 19 | 14 |  |
| Moderate/low | 30 | 25 | 5 |  |
| TNM stage |  |  |  | 0.014 |
| I/II | 35 | 20 | 15 |  |
| III/IV | 28 | 24 | 4 |  |
| Lymphatic metastasis |  |  |  | 0.047 |
| N0 | 38 | 23 | 15 |  |
| N1-3 | 25 | 21 | 4 |  |
