# Supplementary material for: The RBP1–CKAP4 axis activates oncogenic autophagy and promotes cancer progression in oral squamous cell carcinoma
Source: Cell Death Dis. 2020 Jun 25;11(6):488. doi: 10.1038/s41419-020-2693-8 (PMC7316825; doi:10.1038/s41419-020-2693-8)
Supplement: Supplementary file 2 — Supplementary Figure Legends [file 41419_2020_2693_MOESM2_ESM.docx]

**Figure legends**

**Supplemental Fig. S1.** The expression of CKAP4 in OSCC cell lines and HOK cells were measured by qRT-PCR. All data were shown as mean ± SD. *P<0.05, **P<0.01.

**Supplemental Fig. S2.** Potential signaling pathway of the RBP1-CKAP4 axis activates oncogenic autophagy and promotes cancer progression in OSCC.

**Table legends**

**Supplemental** **Table S1.** Correlation between RBP1 expression level and clinicopathological characteristics in OSCC patients
