# Supplementary figures and images for: The RBP1–CKAP4 axis activates oncogenic autophagy and promotes cancer progression in oral squamous cell carcinoma
Source: Cell Death Dis. 2020 Jun 25;11(6):488. doi: 10.1038/s41419-020-2693-8 (PMC7316825; doi:10.1038/s41419-020-2693-8)

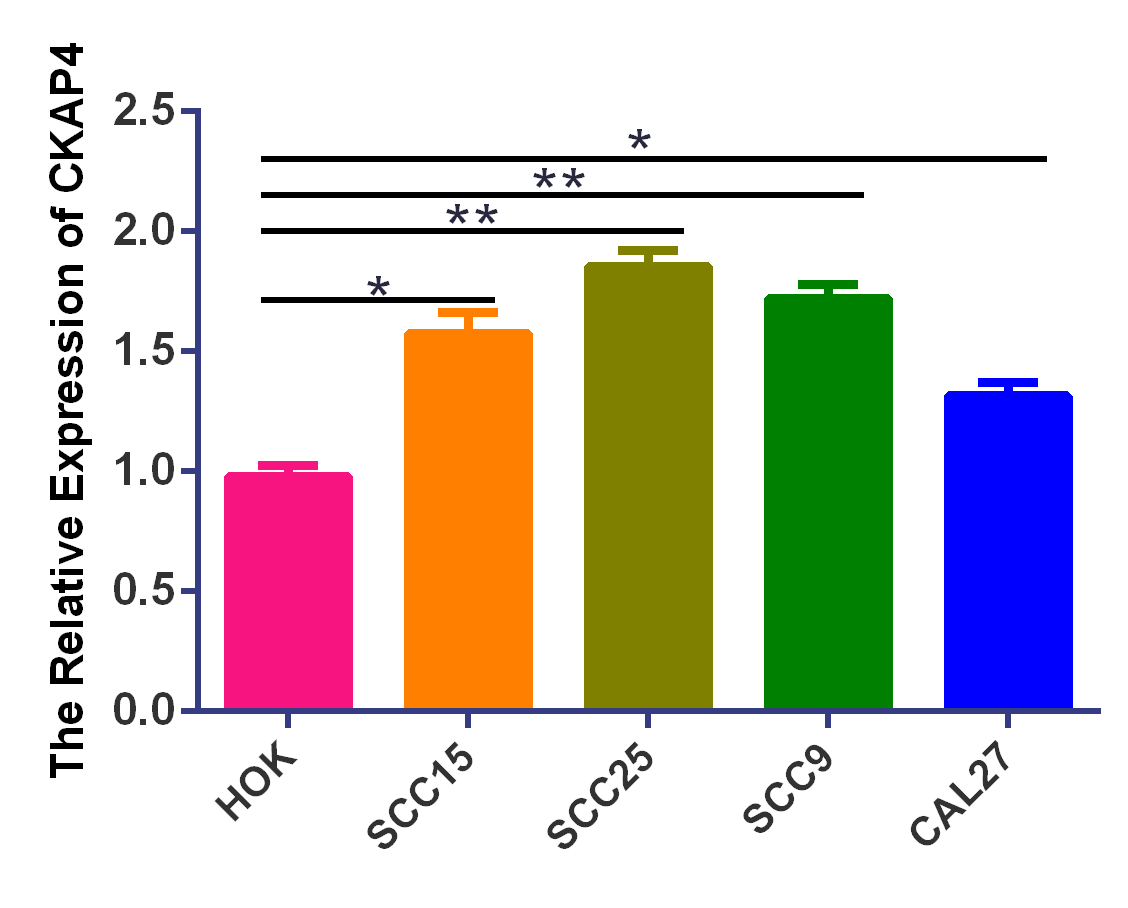

Supplement: Supplementary file 3 — Supplemental Fig. S1 [file 41419_2020_2693_MOESM3_ESM.png]

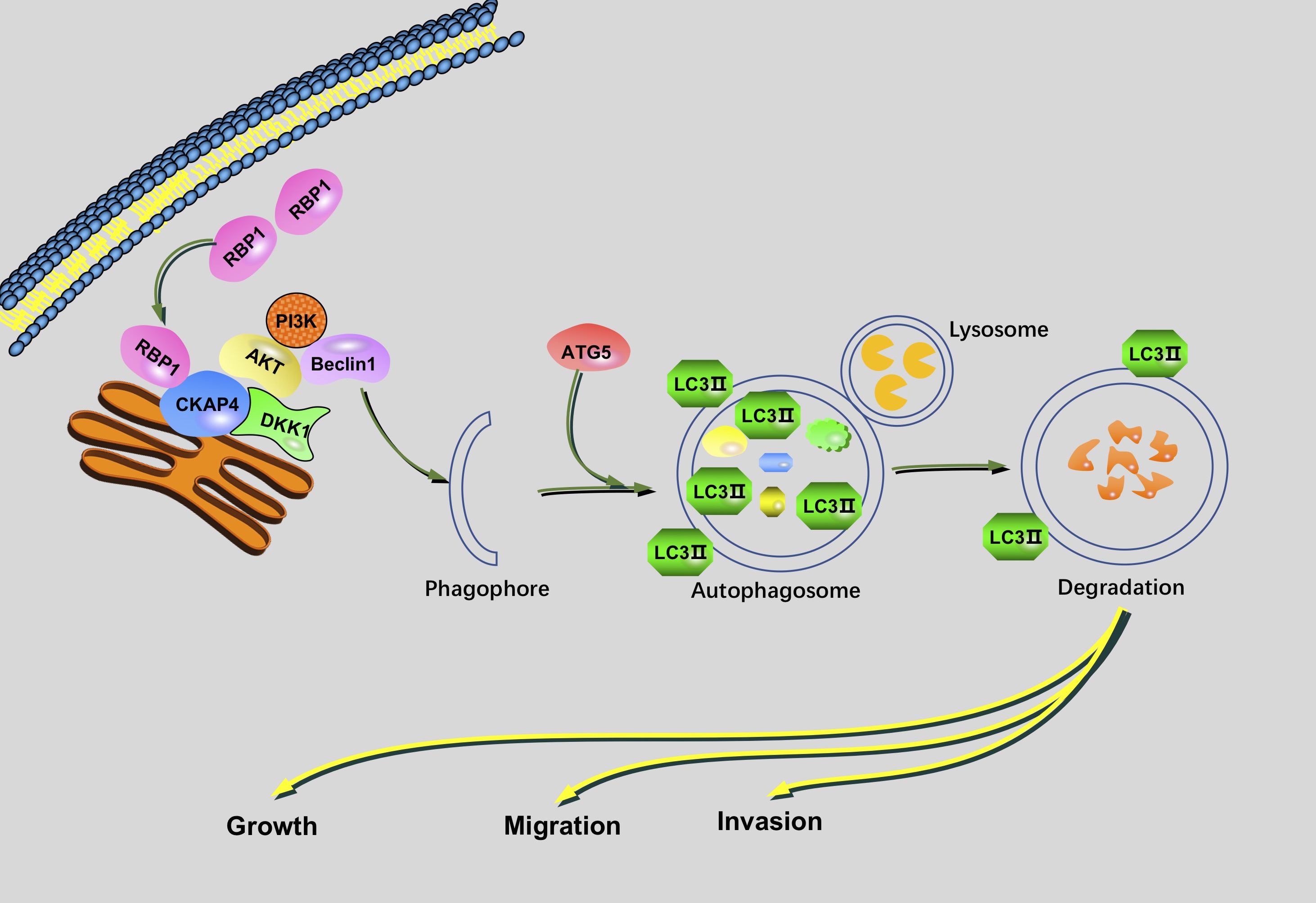

Supplement: Supplementary file 4 — Supplemental Fig. S2 [file 41419_2020_2693_MOESM4_ESM.png]
